# Root Reduction Caused Directly or Indirectly by High Application of Nitrogen Fertilizer Was the Main Cause of the Decline in Biomass and Nitrogen Accumulation in Citrus Seedlings

**DOI:** 10.3390/plants13070938

**Published:** 2024-03-23

**Authors:** Runzheng Niu, Yuan Zhuang, Mohammad Naeem Lali, Li Zhao, Jiawei Xie, Huaye Xiong, Yuheng Wang, Xinhua He, Xiaojun Shi, Yueqiang Zhang

**Affiliations:** 1 College of Resources and Environment, Southwest University, Chongqing 400716, China; niurunzheng@163.com (R.N.); yzhuang48-c@my.cityu.edu.hk (Y.Z.); naeem.lali97@gmail.com (M.N.L.); zhaoli2017@yeah.net (L.Z.); xjwswu@163.com (J.X.); xionghuaye@foxmail.com (H.X.); wyh1996@email.swu.edu.cn (Y.W.); xinhua.he@uwa.edu.au (X.H.); shixj@swu.edu.cn (X.S.); 2 Department of Forestry and Natural Resources, Faculty of Agriculture, Bamyan University, Bamyan 1601, Afghanistan

**Keywords:** excessive nitrogen application, nitrogen uptake, root morphology characteristics

## Abstract

Citrus is the largest fruit crop around the world, while high nitrogen (N) application in citrus orchards is widespread in many countries, which results not only in yield, quality and environmental issues but also slows down the establishment of citrus canopies in newly cultivated orchards. Thus, the objective of this study was to investigate the physiological inhibitory mechanism of excessive N application on the growth of citrus seedlings. A pot experiment with the citrus variety *Orah* (*Orah/Citrus junos*) at four N fertilization rates (0, 50, 100, and 400 mg N/kg dry soil, denoted as N0, N50, N100, and N400, respectively) was performed to evaluate the changes of root morphology, biomass, N accumulation, enzyme activities, and so on. The results showed that the N400 application significantly reduced the total biomass (from 14.24 to 6.95 g/Plant), N accumulation (from 0.65 to 0.33 g/Plant) and N use efficiency (92.69%) in citrus seedlings when compared to the N100 treatment. The partial least squares pathway model further showed that the decline of biomass and N accumulation by high N application were largely attributed to the reduction of root growth through direct and indirect effects (the goodness of fit under the model was 0.733.) rather than just soil N transformation and activity of root N uptake. These results are useful to optimize N management through a synergistic N absorption and utilization by citrus seedlings.

## 1. Introduction

*Citrus* is one of the most important economic crops in the world. Currently, the planting area and yield of citrus in China are increasing in trend, and they are now ranked first in the world [1]. According to the Food and Agriculture Organization survey statistics, China’s citrus planting area was 3.03 × 10^7^ hectares, with a yield of approximately 4.67 × 10^8^ tons in 2021 [2]. Fertilizer, as the “grain” of food, is known as the “life element” of plants. Nitrogen is not only a macromolecular component of plant nucleic acids, proteins, chlorophyll, hormones, and various vitamins, which plays a crucial role in the growth, yield, and quality of citrus trees [3], but also a signaling substance that regulates many plant processes, such as resistance to biotic and abiotic stresses, root development, dormancy, flowering, leaf expansion, seed germination, hormone signaling, and the below-ground traits related to root architecture, etc. [4,5,6]. In particular, NO affects plant root conformation and nutrient acquisition. Previous studies have demonstrated that the regulation of root structure and morphology is partly controlled by the effective free inter-root NO, and that inter-root NO also influences the rate of N cycling between plant and soil through nitrification and denitrification processes [7,8]. Therefore, farmers seek high yields and abundant harvests by applying large amounts of chemical fertilizers, particularly chemical nitrogen (N) fertilizer [9]. However, the negative effects of excessive N application on *citrus* plants are manifested as growth inhibition and metabolic dysregulation in the form of decreased biomass, morphological abnormalities, and reduced growth rates [10]. Previous studies have shown that a nitrogen application of 0.6 kg plant^−1^ significantly increased fruit yield and quality in citrus orchards in China [11]. At the same time, reducing N fertilizer application not only does not reduce yield but also improves *citrus* yield and quality, reduces NO_3_^−^ content in the product which is harmful to consumers [12,13], and also reduces soil and water pollution problems in agricultural areas [14]. Therefore, reducing the application of N fertilizer is essential for *citrus*.

Urea, as one of the most commonly used N fertilizers in the world, cannot be directly absorbed and utilized by plants when applied to soil. It is mainly utilized by crops through urease catalysis and hydrolysis [15]. *Citrus* roots mainly absorb soil NH_4_^+^-N and NO_3_^−^-N for the synthesis of amino acids through various enzymes [16]. Nitrate reductase (NR) is one of the key enzymes in plant N assimilation and root structural remodeling [17], which is responsible for the reduction of NO_3_^−^ to NO_2_^−^ in plant cells [18]. Nitrite reductase is the second enzyme involved in NO_3_^−^ reduction process, which to some extent directly reflects the nutritional status and N assimilation level of plants [19]. Glutamine synthetase and glutamate synthetase are the two main enzymes of NH_4_^+^ assimilation, with glutamine synthetase catalyzing the first step of N assimilation in plant cells [20]. Among them, the activity of nitrate reductase (NR), nitrite reductase (NiR), glutamine synthetase (GS), glutamate synthetase (GOGAT), and other key enzymes involved in N metabolism have been used to evaluate representative biochemical indicators for plant absorption, transportation, and assimilation of N [21]. In recent years, most studies have been conducted on N transformation in citrus orchard soil [22], root N absorption, and assimilation [23,24]. Previous studies have shown that a significant correlation between the soil ammonia oxidation rate and the abundance of ammonia-oxidizing archaea (*AOA*) or ammonia-oxidizing bacteria (*AOB*) was found in the response of acidic red soils to fertilization in southern China [25]. N uptake and the utilization efficiency of citrus seedlings decreased with increasing N applications [13], and either high or low N reduced plant root uptake and root N concentration, destroying plant nutrition and growth [26]; whereas, optimized fertilization promoted root growth and nutrient uptake for healthy and sustainable orchard development [24]. Therefore, the amount of N applied plays an important role in soil N transformation and root N uptake and utilization.

However, there are few reports on the effects of excessive N application on the growth and N accumulation of citrus seedlings. We assumed that excessive N application could be the main factor leading to a decrease in root growth and N accumulation. The aim of this study was to investigate the activity of N metabolism enzymes and related N indicators in plants and soil under unbalanced N application rates in order to clarify the soil N transformation process, *citrus* seedling growth, and N accumulation characteristics for a rational N application to *citrus* seedlings.

## 2. Results

### 2.1. Effects of Different N Application Rates on Soil N Conversion-Related Enzymes and Microorganisms

**Figure 1:**
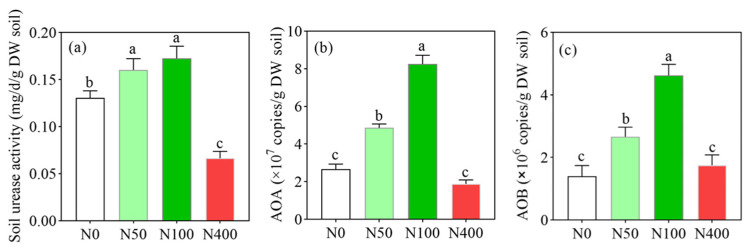
Effects of N fertilization on soil urease activity (**a**), gene copies of *AOA* (**b**), ammonia-oxidizing archaea) and *AOB* (**c**), ammonia-oxidizing bacteria. N0, N50, N100, and N400 represent N fertilization rates at mg/kg dry soil. Data (means ± SD, *n* = 3) followed by different letters represent significant differences between treatments at *p* < 0.05.

Under different N application levels, soil urease activity (Figure 1a) ranged from 0.07 to 0.17 mg d^−1^g^−1^ and showed a trend of first increasing and then decreasing with the increase of N application rates. Compared with the N400 application, N50 and N100 application rates significantly increased soil urease activity by 228.6% and 242.9%, respectively. The gene copies of soil *AOA* (Figure 1b) and *AOB* (Figure 1c) also showed a trend of first increasing and then decreasing with an increase of N applications, and the highest values were 8.25 × 10^7^ and 4.62 × 10^6^ (gene copies/g soil) in the N100 application, respectively. Compared with the N50 and N100 applications, the N400 application significantly inhibited the gene copies of soil *AOA* and *AOB*, which decreased by 77.3% and 62.1%, respectively, compared to the optimized N application. In addition, the gene copy numbers of *AOA* were 10 times than these of *AOB*. Therefore, excessive N application (N400) significantly inhibited soil N transformation-related enzyme activities and microorganisms.

### 2.2. Effects of Different N Application Rates on Soil N Content

**Figure 2:**
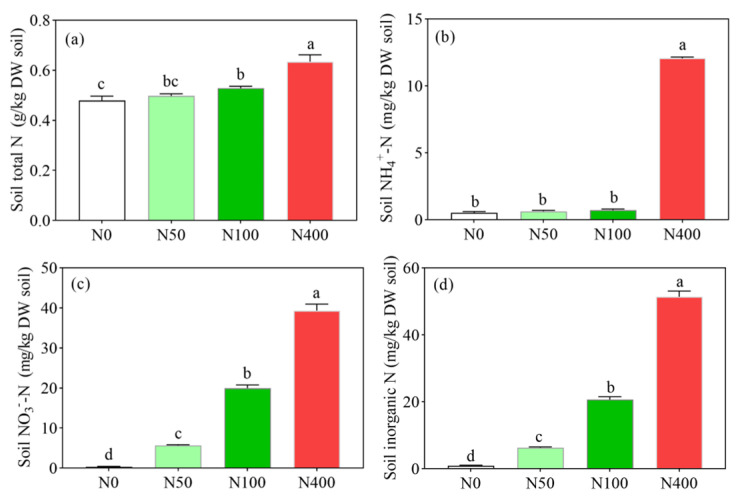
Effects of N fertilization on soil total N content (**a**), soil NH_4_^+^-N content (**b**), soil NO_3_^−^-N content (**c**), and soil inorganic N (**d**). N0, N50, N100, and N400 represent N fertilization rates at mg/kg dry soil. Data (means ± SD, *n* = 3) followed by different letters represent significant differences between treatments at *p* < 0.05.

Soil total N, NH_4_^+^-N, NO_3_^−^-N, and soil inorganic N all increased with the increase of N application rates. Soil N was the lowest at 0.48 k/kg under N0 (Figure 2a), 0.53 mg/kg under N0 (Figure 2b), 0.37 mg/kg under N0 (Figure 2c), and 0.90 mg/kg under N0 (Figure 2d), respectively. Soil total N, NH_4_^+^-N, NO_3_^−^-N, and soil inorganic N content were the highest at 0.63 k/kg (Figure 2a), 12.05 mg/kg (Figure 2b), 39.34 mg/kg (Figure 2c), and 51.40 mg/kg (Figure 2d), respectively. Compared with the N100 application, the N400 application significantly increased the content of soil total N, NH_4_^+^-N, NO_3_^−^-N, and soil inorganic N, increasing by 21.1% (Figure 2a), 1573.6% (Figure 2b), 97.2% (Figure 2c), and 148.6% (Figure 2d), respectively. Compared with other N treatments, the N400 application significantly increased the soil NH_4_^+^-N and NO_3_^−^-N contents, which were 16.74 (Figure 2b) and 1.97 (Figure 2c) times higher than those under the N100 application, respectively.

### 2.3. Effects of Different N Application Rates on Root N Content

**Figure 3:**
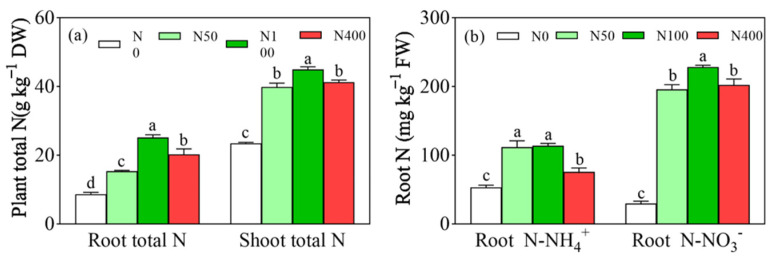
Effects of N fertilization on plant total N (**a**) and root N (**b**). N0, N50, N100, and N400 represent N fertilization rates at mg/kg dry soil. Data (means ± SD, *n* = 3) followed by different letters represent significant differences between treatments at *p* < 0.05.

Under different N fertilization levels, root total N (Figure 3a), shoot total N (Figure 3a), root NH_4_^+^-N (Figure 3b), and NO_3_^−^-N (Figure 3b) showed a trend of first increasing and then decreasing with an increase of N applications in the range of 8.66~25.13 g kg^−1^, 23.47~44.96 g kg^−1^, 75.53~113.54 mg kg^−1^, and 195.91~228.41 mg kg^−1^, respectively, and the shoot total N content was greater than root total N (Figure 3a). Compared with the N400 application, the N100 application increased root and branch total N by 24.10% and 8.86%, respectively (Figure 3a). The N100 treatment had the highest root total N, while the lowest root total N was under N0 (Figure 3b). Except for N0, root NO_3_^−^-N had higher values than all other N fertilization rates, compared to root NH_4_^+^-N (Figure 3b). Compared with the N100 application, the N400 application significantly inhibited root absorption of NH_4_^+^-N and NO_3_^−^-N by 33.48% and 11.44%, respectively (Figure 3b). Compared with the N50 application, the N100 application significantly increased root NO_3_^−^-N from 195.91 to 228.42 mg/kg (Figure 3b). Compared with the N0, root NH_4_^+^-N was significantly increased under all other N fertilization rates, while there was no significant root NH_4_^+^-N difference between N50 and N100 (Figure 3b). Thus, excessive N application can decrease root N content.

### 2.4. Effects of Different N Application Rates on Enzymes Related to Root N Transformation

**Figure 4:**
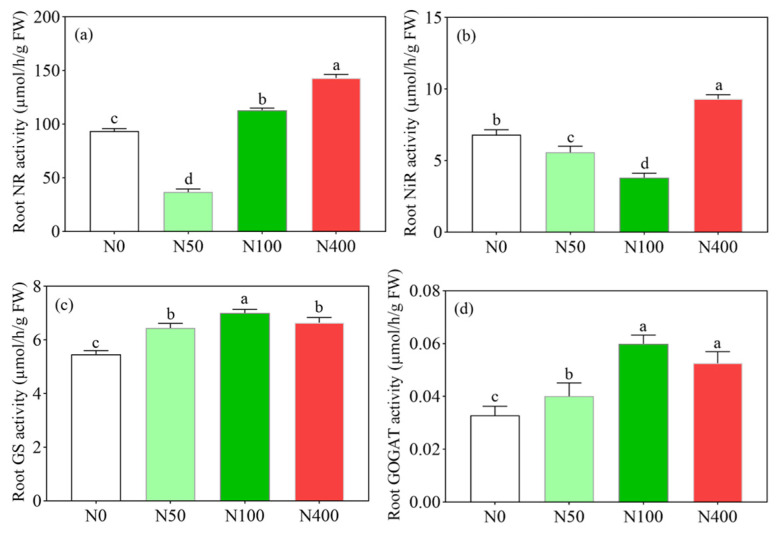
Effects of N fertilization on nitrate reductase activity ((**a**), NR), nitrite reductase activity ((**b**), NiR), glutamine synthetase activity ((**c**), GS), and glutamate synthase activity ((**d**), GOGAT). N0, N50, N100, and N400 represent N fertilization rates at mg/kg dry soil. Data (means ± SD, *n* = 3) followed by different letters represent significant differences between treatments at *p* < 0.05.

Under different N applications, root NR and NiR activities showed a trend of first decreasing and then increasing with the increase of the N rate (Figure 4a,b), while root GS and GOGAT activities showed a trend of first increasing and then decreasing with the N rate (Figure 4c,d). There were significant differences in root NR (Figure 4a) and NiR (Figure 4b) activities among different N applications. Compared with the N100 application, the N400 application significantly increased the activity of root NR (Figure 4a) and NiR (Figure 4b) by 143.16 and 9.31 µmol/h/g, respectively, which were 26.57% and 144.36% higher than the N100 application treatment. Among the root GS and GOGAT activities, the N100 application treatment had the highest GS and GOGAT activities at 7.01 and 0.060 µmol/h/g, respectively. Root GS and GOGAT activities were lowest at 5.47 and 0.03 µmol/h/g, under N0, respectively. Compared with the N100 application, the N400 application inhibited the enzyme activity of root GS and GOGAT.

### 2.5. Effects of Different N Application Rates on the Development of Citrus Root Systems

Under different N applications, the total root length, root surface area, root volume, and total biomass of citrus seedlings showed a trend of first increasing and then decreasing with an increase of the N rate (Table 1). Among them, the total root length, root surface area, root volume, total root length, and total root biomass were the highest under the N100 application treatment. Compared with the N100 application, the N400 application significantly inhibited total root length, root surface area, root volume, and total root biomass, reducing them by 48.08%, 43.27%, 43.52%, and 51.19%, respectively. Among various N rates, there was no significant difference in total root length, root surface area, root volume, and total biomass between N0 and N50, while there was a significant difference between other N treatments.

**Figure 5:**
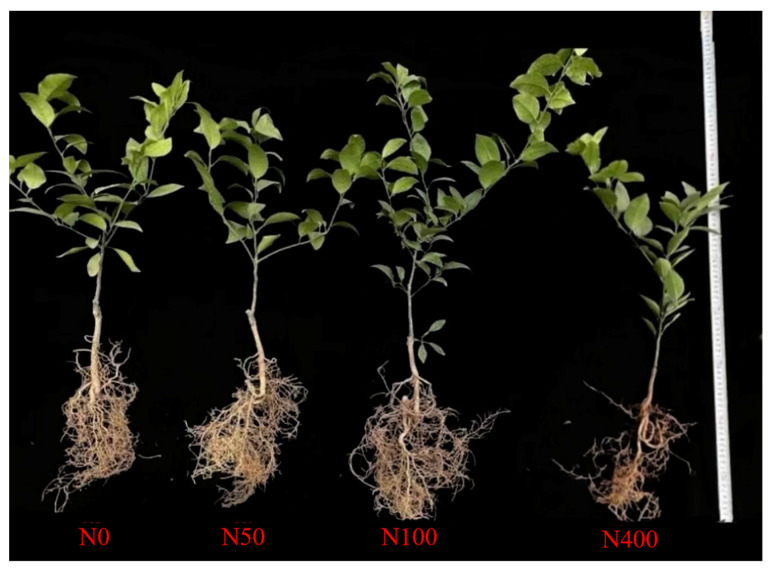
Growth and development of *citrus* seedlings under different N levels.

There were significant differences in the growth of citrus seedlings under different N application rates (Figure 5). Compared with N50 and N100 treatments, under N0 and N400 root growth was limited as the root system was sparse and short, and the overall root length was short. Among them, the growth of *citrus* seedlings was the worst under N400, while the best under N100.

### 2.6. Effects of Different N Application Rates on the Growth of Citrus Seedlings

Under different N rates application, the shoot biomass trend of *citrus* seedlings in each treatment was N100 > N50 > N0 > N400, and the biomass trend of various organs in *citrus* seedlings was root > leaf > branch (Table 2). The biomass and root-to-shoot ratio of various parts of *citrus* seedlings showed a trend of first increasing and then decreasing with an increase of the N rate. Compared with the N100 application, the N400 application significantly reduced biomass production in roots, branches, and leaves, which were 51.19%, 20.75%, and 33.42% lower than with N100, respectively. Compared with the N0 application, both the N50 and N100 applications increased the biomass production of various organs in *citrus* seedlings. Among them, there was a significant difference in biomass production between different parts of citrus seedlings treated with the N100 application and the N0 application. Under various N application levels, the root-to-shoot ratio was lowest at 0.78 under the N400 application treatment and highest at 1.12 under the N100 application treatment.

### 2.7. Effects of Different N Fertilizer Application Rates on N Uptake and Utilization Efficiency of Citrus Seedlings

Under different N rates, the total N accumulation ranked as N100 > N50 > N400 > N0 (Table 3). Between different organs, N concentrations in citrus seedlings were leaf > root > branch. While the total N content in different organs was root > leaf > branch. Total N concentration and N accumulation between *citrus* organs showed a trend of first increasing and then decreasing with an increase of N rates. Compared with the N100 application, the N400 application significantly reduced the total N concentration and N accumulation in the different organs of *citrus* seedlings. Compared with the N100 application, the total N accumulation in the N400 application treatment decreased by 49.23%. Compared with the N50 application, there was no significant difference in the N accumulation in the different citrus organs, but there was in the whole seedling under the N400 application. Among the various N rates, the highest N fertilizer utilization rate was under N100 at 56.60%, and the lowest was 4.14% under N400, indicating a 13.67 times higher fertilizer utilization rate under the N100 application treatment than under the N400 application treatment.

### 2.8. Correlation Analysis and Principal Component and Partial Least Squares Path Analysis of N Absorption-Related Indicators

**Figure 6:**
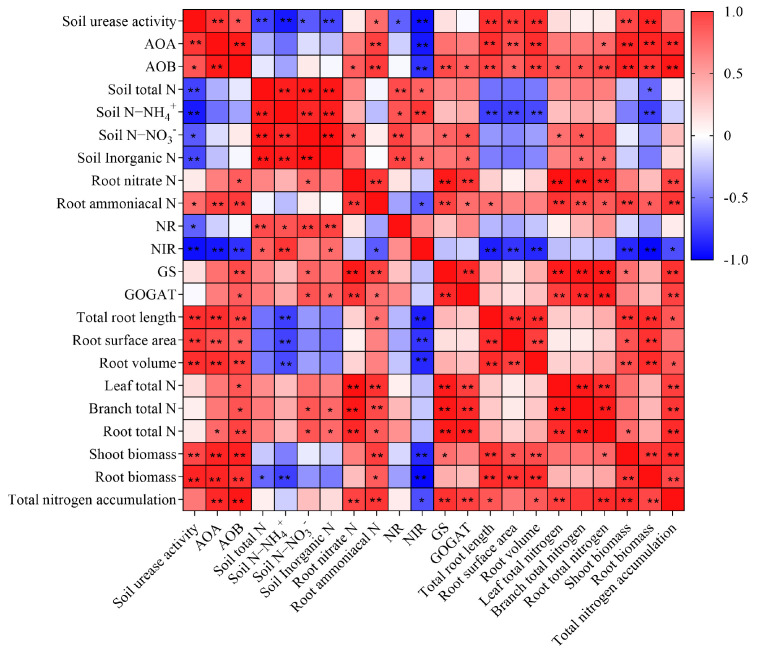
Pearson correlation coefficient heat map of N uptake indicators in *citrus* seedlings. Red color indicates a significant positive correlation, and blue color indicates a significant negative correlation. The significance levels were set as follows: *p* < 0.05 (*), 0.05 < *p* < 0.01 (**), and (*n* = 12). NR, nitrate reductase; NiR, nitrite reductase; GS, glutamine synthetase; GOGAT, glutamate synthase.

There was a significant correlation between soil N and root nitrogen transformation-related enzymes activities, as well as between soil nitrogen transformation-related microbial and enzyme activities and root morphological characteristics (Figure 6). Soil total N, NH_4_^+^-N, NO_3_^−^-N, or inorganic N is significantly positively correlated with metabolic indicators such as root NR and NiR activity. In addition, there was a significantly positive correlation between soil urease, *AOA* or *AOB*, and total root length, root surface area, root volume, aboveground biomass, and root biomass production.

**Figure 7:**
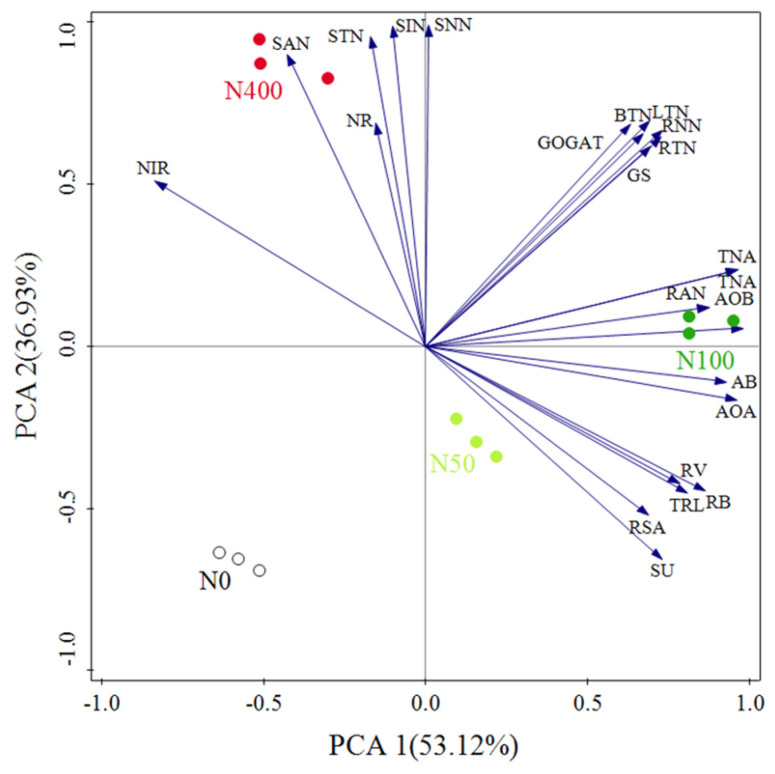
Principal component analysis results of N absorption indicators in *citrus* seedlings. Note: SU: Soil urease; STN: Soil total N; SAN: Soil NH_4_^+^−N; SNN: Soil NO_3_^−^−N; SIN: Soil Inorganic N; RNN: Root nitrate N; RAN: Root ammoniacal N; TRL: Total root length; RUA: Root surface area; RV: Root volume; LTN: Leaf total N; BTN: Branch total N; RTN: Root total N; AB: Aboveground biomass; RB: Root biomass; TNA: Total N accumulation.

Principal component analysis (PCA) was used to compare the similarity of N absorption-related indicators in *citrus* seedlings under different N rates. The explanatory values of the first and second principal component axes for *citrus* N absorption indicators were 53.12% and 36.93%, respectively (Figure 7). On the N level, according to the PCA1 axis, the order ranked as N100 > N50 > N400 ≈ N0, indicating that N absorption-related indicators changed significantly with an increase of the N rate and N100 had the most significant impact on N absorption indicators. Among the principal components of PC1, the positive load weights of soil *AOA*, *AOB*, total N accumulation, and root biomass were highest, while the negative load weights of root NiR and soil ammonium N were highest. Among the principal components of PC2, the positive load weights of soil total N, ammonium N, nitrate N, and inorganic N were highest, while the negative load weights of soil urease, root surface area, and root biomass were highest. Based on this, the above indicators can be used as the core indicators for nitrogen absorption in *citrus*.

**Figure 8:**
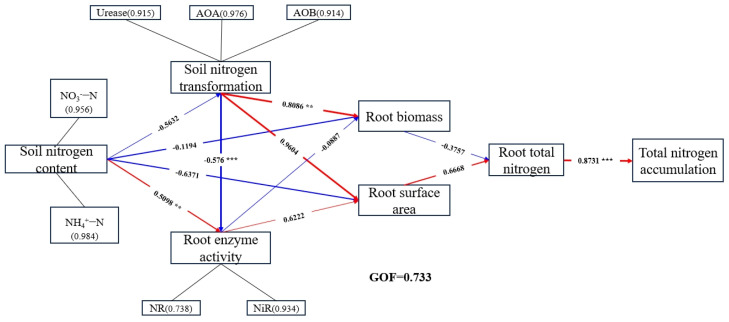
Partial least squares path model (PLS−PM) analysis of core indicators of N absorption in *citrus* under different treatments. Color represents positive and negative correlation (red represents positive correlation, blue represents negative correlation). The asterisk represents the magnitude of the correlation (** represents significance at *p* < 0.01, and *** represents significance at *p* < 0.001). The numbers in parentheses correspond to specific load values.

Finally, the relationships among the core indicators of *citrus* N uptake (soil N, soil N transformation-related microbes and enzyme activities, root N metabolizing enzyme activities, root surface area and root biomass, and root total N and plant total N accumulation) were analyzed under different N rates using the partial least squares path model (PLS-PM). The overall fit of the model was GOF = 0.733 (Figure 8). Studies have shown that microbial and enzyme activities related to soil N transformation and enzyme activities related to root N metabolism have a significant effect on root growth; the soil nitrogen content had a direct negative effect on root biomass (λ = −0.1194) and surface area (λ = −0.6371). As soil nitrogen increased, it affected root enzyme activities through soil nitrogen transport-related enzymes and microorganisms (λ = −0.576, *p* < 0.001) and indirectly affected root biomass production and N accumulation. These processes can directly or indirectly affect N uptake by roots and thus N accumulation in the plant organs.

## 3. Discussion

### 3.1. Effects of N Application on Soil N Transformation and Supply

Soil enzyme activity reflects the amount of soil nutrients, and it is one of the important indicators for evaluating soil fertility [27]. The results of this study showed that soil urease activity increased and then decreased with the increase of N applications, in which the N50 and N100 applications increased soil urease activity, and the N400 application significantly decreased soil urease activity. This is consistent with the changing pattern of urease activity and diversity of urea bacterial community under long-term urea applications [28]. And compared with the control N0 treatment, soil urease activity under excess N applications was lower [29], which is consistent with the results of this study because excess nitrogen application led to an increase in soil NO_3_^−^-N accumulation, and a high NO_3_^−^-N concentration could lead to a decrease in the activities of protease and urease [30].

Soil microorganisms, as an important part of the soil ecosystem, play a crucial role in maintaining soil fertility, improving soil structure, and decomposing organic matter and minerals that are not easily absorbed by plants [31]. Soil *AOA* and *AOB* not only play an important role in global N cycling, but also their catalyzed ammonia oxidation process is a first and rate-limiting step of the nitrification process [32]. In acidic agricultural soils, the abundance of soil *AOB* was significantly lower than that of *AOA* [33,34], and the application of urea significantly increased the abundance of soil for both *AOA* and *AOB* genes, with a higher *AOA* abundance [35]. These are consistent with our findings, and the reason may be that long-term N applications led to an increase in soil NH_4_^+^-N and NO_3_^−^-N concentrations [36], which stimulated soil nitrification and consequently increased the population of soil *AOA* and *AOB* [37]. In this study, total soil N, NH_4_^+^-N, NO_3_^−^-N and soil inorganic N increased with increasing N applications. This is consistent with previous studies on *potato* [38], *corn* [39], and *tomato* [40], where soil N content was significantly and positively correlated with N applications and increased with N applications. It was found that soil *AOB* gene abundance tended to increase and then decrease with the increase of N fertilizer applications, with the highest number at the N100 rate. However, with the increase of N application, the number of soil *AOA* gene copies decreased significantly, but these values were higher than the number of *AOB* gene copies [41], which were consistent with the N response of soil *AOB*, but not soil *AOA* in this study. The reason for this difference might be due to different soil types and N rates. In addition, soil type is a major determinant of *AOA* community structure [42], while soil *AOB* and *AOA* can have different growth patterns under different soil N conditions in the same soil type [43].

### 3.2. Effects of N Application on N Uptake and Transport-Related Indicators in Citrus Roots

The activities of N-assimilating enzymes play a crucial role in maintaining plant growth and development. Previous studies have shown that N supply can increase the activity of key enzymes involved in N metabolism [44,45], e.g., low N resulted in enhanced NR activity, but high N caused a decrease in NR activity [17]. These were consistent with the results of the present study that NR and NiR activities were higher in roots under N0 than under the N50 application, while GS and GOGAT showed a trend of increasing and then decreasing with an increasing N rate, and that N400 significantly inhibited their activities. The reason is that the activity of root GS/GOGAT gradually increased with the increase of NH_4_^+^ concentration, and when the NH_4_^+^ concentration exceeded 3 mM, it would inhibit the NH_4_^+^-induced increase of GS/GOGAT activity [46], which caused the unique GS/GOGAT pathway in the plant tissues to stop synchronizing its action [47], and thus caused the GS and GOGAT activities to be reduced under excess N. The difference with the trends of NR and NiR might be due to the fact that excessive N application stimulated the activities of root NR and NiR although it inhibited the activities of GS/GOGAT pathways. This is consistent with the results that appropriately increasing N levels can increase the activities of N-assimilating enzymes such as NR and GS to reach a synchronous increase, and that excessive N levels can increase NR but decrease the activity of GS [48]. Meanwhile, high N stress also leads to the down-regulation of gene expressions of NR, NiR, GS, and GOGAT [49]. Studies in yellow fruit *citrus* demonstrated that a moderate increase in N fertilization significantly increased the activities of key N metabolizing enzymes (NR, NiR,) and the expression of their related genes in roots, leaves, and fruits [23].

In summary, a moderate amount of N increased the activities of key enzymes for N metabolism in roots, while excessive N inhibited their enzymatic activities. The above results are also further demonstrated in Figure 3. Root NH_4_^+^-N and NO_3_^−^-N were high under N100, while they were significantly reduced under N400.

### 3.3. Effects of N Application on the Root Morphology of Citrus

The plant root system is the only one in direct contact with the soil and has a variety of important physiological functions such as absorption, synthesis, secretion, and sensing and plays a crucial role in crop yield, and any environmental factors and cultivation measures affecting root growth will affect the growth and development of the whole plant [50,51,52]. Nitrogen is known as the “life element”, which is involved in the composition of a variety of metabolic and active substances in plants, and a lack of N can lead to weakened root growth, altered root structure, reduced plant biomass, and reduced photosynthesis. In contrast, high N inhibits the elongation of primary roots and the formation of lateral roots [48], and this growth inhibition will lead to a reduction in the biomass and root–crown ratio of *citrus* [53], which in turn will result in the growth inhibition and metabolic dysfunction of *citrus* plants, which is manifested as a decrease in biomass, morphological abnormalities, and a reduction in growth rate [54]. In addition, excessive N application may cause ammonium toxicity phenomena, such as reduced plant biomass, altered root conformation, a decreased root–crown ratio, and leaf chlorosis [10]. The results of this study show that the root growth of *citrus* seedlings differed significantly under different N rates. Under the N400 application, the root growth was limited as sparse and short, and was best under the N100 application. Meanwhile, the root length, root surface area, root volume, root–crown ratio, and biomass of all organs, total N concentrations, and N accumulations of citrus seedlings showed a tendency to first increase and then decrease with the increase of N rates. These were in agreement with the results of previous studies on *spruce* [55], *cotton* [56], and *passion fruit* [57]. The idea was further supported that the root dry weight, root length, and root surface area of rice were increased with an increase of N within a certain range of N applications [58]. However, when excess N is applied, it significantly inhibits *corn* root elongation and leads to a reduction in root dry weight [59]. This is due to the fact that high nitrate levels in the buds inhibit starch synthesis, which in turn reduces root sugar levels [60]. This study found through principal component analysis (PCA) and the partial least squares path model (PLS-PM) that N fertilizer application into soil directly or indirectly affects root biomass and surface area through soil N transformation-related microorganisms and enzyme activities, as well as root N assimilation-related enzyme activities, thereby causing a significant impact on the total nitrogen accumulation of plants. It can be inferred that under high-nitrogen conditions in the acidic red soil areas of southern China, the nitrogen assimilation and utilization ability of *citrus* plants directly affects their growth and development [61,62]. Previous studies also showed that excessive nitrogen application suppressed the expression of nitrogen genes related to nitrogen transport and genes for assimilation-related enzyme activities in the root system [10,23]. Therefore, an excessive application of nitrogen fertilizer can inhibit root growth.

In summary, the N400 application inhibited the root growth of *citrus* seedlings, which in turn led to a reduction of the root–shoot ratio, plant biomass production, and N accumulation. In contrast, the N100 application promoted the growth of *citrus* seedlings and increased plant N uptake. In terms of N fertilizer utilization, the N100 application treatment had the highest N utilization rate at 56.6%, followed by the N50 application, and the N400 application treatment significantly inhibited plant N uptake of *citrus* seedlings.

## 4. Materials and Methods

### 4.1. Experimental Location

The pot experimental site was conducted at the Nation Purple Soil Fertility and Fertilizer Effect Monitoring Base at the Southwest University campus (30°26′31″ N and 106°26′45″ E) in Beibei District, Chongqing, southwest China, which has a subtropical monsoon humid climate. The soil is classified as a Eutric Regosol [63]. According to the United States Department of Agriculture (USDA) soil taxonomy, the soil belongs to entisol [64]. It is within a typical hill area with an elevation of about 385 m, a mean annual rainfall of 400–500 mm, and a mean annual temperature of 15–22 °C.

### 4.2. Experimental Design

A total of 60 one-year-old citrus seedlings of *fertile orange* (*Orah*) grafted on *fragrant orange* (*Citrus junos Sieb. ex Tanaka*) rootstocks were planted in March 2021 in plastic pots. The 60 citrus seedlings were each transplanted into pots (height 30 cm, diameter 25.5 cm) filled with 8 kg of purple soil. (The soil is classified as a Eutric Regosol [63], see its basic physiochemical properties in Table 4 [65]). Four nitrogen (N, urea) fertilization treatments were applied as (1) N0, zero-N control; (2) N50, 50 mg N/kg DW soil; (3) N100, 100 mg N/kg DW soil; and (4) N400, 400 mg N/kg DW soil [66]. A total of four N-level treatments with 15 replicates per treatment and a total of 60 pots (1.5 m apart from each other) were completely randomized and arranged in the experimental site. (Nation Purple Soil Fertility and Fertilizer Effect Monitoring Base at the Southwest University campus.) Among them, phosphorus and potash fertilizers were used as basal fertilizers mixed with soil in plastic pots. Calcium superphosphate was used for phosphorus at a rate of 100 mg kg^−1^, and potassium sulfate was used for potassium at a rate of 50 mg kg^−1^ [67]; whereas, between March and October 2021, urea was sprayed uniformly, dissolved in water, over 14 applications to the soil surface. (Fertilizer was applied every two weeks at the same rate for each application.) The rest would be managed according to conventional *citrus* planting practices, and the trial ended in March 2022.

### 4.3. Determination of Total N and Root Morphology in Plant Roots, Branches, and Leaves

Six pots were sampled for each treatment, with three pots for physiological determination and the other three pots for biomass determination, sampled separately as leaves, branches, main roots, and lateral roots on 15 March 2022. The harvested plant tissues were oven-dried at 65 °C for 72 h and ground into 1 mm powder which was digested with H_2_SO_4_-H_2_O_2_; then, N concentrations were determined using the Kjeldahl method. The remaining fresh plants and soil samples were quickly frozen in liquid N and stored in a −80 °C refrigerator for the subsequent analysis of other parameters. Data of root volume, root length, and root surface area were first scanned using a root scanner EPSON (Expression 10000XL 1.0, Epson Inc., Suwa City, Japan) and then analyzed using WinRHIZO Pro (S) v. 2004b software (Rcgcnt Instrument cnt Inc., Québec City, QC, Canada).

Total biomass (g tree^−1^) = shoot biomass + root biomass.

Root-to-shoot ratio = root biomass/shoot biomass.

Amount of N accumulation of each organ (g tree^−1^) = N concentration × total dry mass of each organ.

N utilization efficiency (%) = (N accumulation under N application treatment − N accumulation under no N application treatment)/amount of N applied × 100%.

### 4.4. Determination of Soil Urease, Ammonia Nitrate N, Root Enzyme Activity, and Ammonia Nitrate N

Soil ammonium and nitrate N were analyzed and measured using a flow analyzer. Soil urease (EC3.5.1.5), other enzyme activities, root ammonium, and nitrate N were measured using commercial test kits (Comin Biotechnology Co., Ltd., Suzhou, China) according to the manufacturer’s instructions. The measured enzymes included nitrate reductase (NR, EC1.7.1.3), nitrite reductase (NiR, EC1.7.1.15), GS glutamine synthetase (EC6.3.1.2), and GOGAT glutamic acid synthetase (EC1.4.1.14) [68]. Fresh citrus roots were ground at 4 °C, and the activities of different enzymes were determined with commercial assay kits. NR and GOGAT activity of roots were extracted and determined according to the method described by Li et al., and their activity was represented by an absorbance value at 340 nm [69]. NiR and GS activity of roots was extracted and determined according to the method described by Zahoor et al., and their activity was represented by an absorbance value at 540 nm [70].

### 4.5. Determination of Soil AOA and AOB

Soil DNA was extracted using PowerSoil^®^ DNA isolation kits (MoBioInc., Carlsbad, CA, USA), following the manufacturer’s instructions. Extracted DNA was quantified and checked for purity with a Nanodrop 1000 Spectrophotometer (Thermo Fisher Scientific, Waltham, MA, USA) and by electrophoresis using a 1.5% agarose gel. Extracted DNA was stored at −20 °C [25].

Real-time quantitative PCR (qPCR) was employed to quantify the abundance of functional genes using primers published in previous studies (Table 5) in a Fluidigm BioMark HD™ System [71]. Reaction volumes for qPCR were 20 μL and contained 10 μL of EvaGreen Master Mix (Qiagen, Germantown, MD, USA), 1 μL each of forward and reverse primer from stock solutions (10 μM), and 2 μL of DNA template (5 ng/μL). A program with an initial 3 min at 95 °C followed by 40 cycles at 95 °C for 5 s and 57 °C for 20 s and 72 °C for 30 s was applied for the PCR.

### 4.6. Data Analysis

The experimental data (means ± SD, *n* = 3) were analyzed by one-way analysis of variance (ANOVA) and the Tukey (*p* < 0.05) significant difference method using IBM SPSS 26.0, and Pearson’s correlation of citrus nitrogen uptake-related indices using SPSS software, with *p* < 0.05 indicating significant differences and *p* < 0.01 indicating highly significant differences. Principal component analysis (PCA) for the relationships among the soil N and its related microorganisms and enzyme activities, enzyme activity related to root N assimilation and N content, and N accumulation in the various organs of plants were performed using Canoco 5.0 software. The partial least squares path model (PLS-PM) was used to demonstrate cause and effect relationships among the observed and latent variables. The estimates of path coefficients and the significance *p*-value in the path model were validated by R software (v. 4.3.2) using the “plspm” and “vegan” package. The above data plots were drawn using GraphPad Prism 9.5.1 (San Diego, CA, USA) and PowerPoint 2019 (Microsoft, Redmond, WA, USA) software.

## 5. Conclusions

Soil N content ranged from 0 to 400 mg N/kg; DW soil increased with the increase of N fertilization rates. Although the N400 application stimulated NR and NiR activities in the roots, the N100 application significantly increased soil urease activity (162.12%), soil *AOA* (337.36%) and *AOB* (164%) abundance, plant total N accumulation (96.97%), shoot biomass (39.17%), root morphological characteristics (root length (48.76%), root surface area (76.28%), and root volume (77.06%)), and root nitrogen assimilating enzyme (GS (5.52%) and GOGAT (13.21%)) activities, as compared to the N400 treatment. Finally, the analysis based on the partial least squares path model (PLS-PM) shows that the soil nitrogen content had a direct negative effect on root biomass (λ = −0.1194) and surface area (λ = −0.6371); as soil nitrogen increased, it affected root enzyme activities through soil nitrogen transport-related enzymes and microorganisms (λ = −0.576, *p* < 0.001) and indirectly affected root biomass production and N accumulation. These processes can directly or indirectly affect N uptake by roots and thus N accumulation in the plant organs. Overall, the N100 mg N/kg dry soil was more suitable for the expression of enzymes related to N uptake and metabolism in *citrus* roots, and thus plant growth.

## Figures and Tables

**Table 1:** Effects of different N levels on total root length (cm), root surface area (cm^2^), root volume (cm^3^), and total biomass (g) of citrus seedlings. Data (means ± SD, *n* = 3) followed by different letters represent significant differences between treatments at *p* < 0.05.

	Total Root Length	Root Surface Area	Root Volume	Total Biomass
mg/kg	cm	cm²	cm³	g/Plant DW
N0	2369 ± 180.40 ab	477 ± 110.24 ab	7.68 ± 1.10 b	9.88 ± 0.77 b
N50	2525 ± 245.79 ab	494 ± 60.36 ab	7.85 ± 0.57 b	11.47 ± 0.48 b
N100	3222 ± 249.72 a	587 ± 49.73 a	10.11 ± 0.61 a	14.24 ± 1.03 a
N400	1673 ± 384.24 c	333 ± 68.13 b	5.71 ± 0.92 c	6.95 ± 0.99 c

**Table 2:** Effects of different N levels on leaves, branches, roots, shoots, and the root/shoot relationship of *citrus* seedlings. Data (means ± SD, *n* = 3) followed by different letters represent significant differences between treatments at *p* < 0.05.

Treatment	Leaf	Branch	Root	Shoot	Root/Shoot Ratio
mg/kg	g/Plant DW	g/Plant DW	g/Plant DW	g/Plant DW	g/Plant DW
N0	5.54 ± 0.74 b	3.90 ± 0.79 b	9.88 ± 0.77 b	9.35 ± 0.17 c	1.06 ± 0.08 ab
N50	5.86 ± 0.49 b	4.97 ± 0.44 ab	11.47 ± 0.48 b	10.83 ± 0.72 b	1.06 ± 0.03 ab
N100	7.42 ± 0.89 a	5.30 ± 0.63 a	14.24 ± 1.03 a	12.72 ± 0.31 a	1.12 ± 0.07 a
N400	4.94 ± 0.49 b	4.20 ± 0.74 ab	6.95 ± 0.99 c	9.14 ± 1.22 c	0.78 ± 0.22 c

**Table 3:** Effects of different N levels on total N and N accumulation in roots, stems, and leaves of *citrus* seedlings and N use efficiency (NUE) in plants. Data (means ± SD, *n* = 3) followed by different letters represent significant differences between treatments at *p* < 0.05.

Treatment	Root Total N and N Accumulation		Stem Total N and N Accumulation		Leave Total N and N Accumulation		Total Plant N Accumulation	NUE
mg/kg	g/kg DW	g/Plant DW	g/kg DW	g/Plant DW	g/kg DW	g/Plant DW	g/Plant DW	%
N0	8.66 ± 0.52 d	0.09 ± 0.11 c	8.19 ± 0.31 d	0.03 ± 0.01 c	15.28 ± 0.18 d	0.08 ± 0.01 c	0.2 ± 0.01 d	-
N50	15.30 ± 0.25 c	0.18 ± 0.01 b	14.51 ± 0.69 c	0.07 ± 0.01 b	25.34 ± 0.49 b	0.15 ± 0.01 b	0.4 ± 0.02 b	48.90
N100	25.13 ± 0.79 a	0.36 ± 0.06 a	18.04 ± 0.12 a	0.10 ± 0.01 a	26.92 ± 0.78 a	0.20 ± 0.03 a	0.65 ± 0.05 a	56.60
N400	20.25 ± 1.58 b	0.14 ± 0.03 b	16.27 ± 0.60 b	0.07 ± 0.01 b	25.03 ± 0.29 c	0.12 ± 0.01 b	0.33 ± 0.02 c	4.14

**Table 4:** Basic physiochemical properties of the test soil.

pH Soil:Water = 1:2.5	Bulk Density	OM	TN	NO_3_^−^-N	NH_4_^+^-N	Olsen-P	Available K
	g/cm³	g/kg	g/kg	mg/kg	mg/kg	mg/kg	mg/kg
5.47	1.43	8.82	0.57	12.5	2.95	37.4	161

Note: OM: Organic matter; TN: Total N.

**Table 5:** Primers for analyzing soil *AOA* and *AOB* expression.

Functional Genes	Primers	Primer Sequence	Fragment Length
amoA	amoAF	5′-STAATGGTCTGGCTTAGACG-3′	600 bp
	amoAR	5′-GCGGCCATCCATCTGTATGT-3′	
bamoA	bamoA-1F	5′-GGGGTTTCTACTGGTGGT-3′	491 bp
	bamoA-2R	5′-CCCCTCKGSAAAGCCTTCTTC-3′	

## Data Availability

The data that support the findings of this study are available from the corresponding author (Yueqiang Zhang) upon reasonable request.
